# A simple, reproducible and cost-effective procedure to analyse gut phageome: from phage isolation to bioinformatic approach

**DOI:** 10.1038/s41598-019-47656-w

**Published:** 2019-08-05

**Authors:** Camille d’Humières, Marie Touchon, Sara Dion, Jean Cury, Amine Ghozlane, Marc Garcia-Garcera, Christiane Bouchier, Laurence Ma, Erick Denamur, Eduardo P.C.Rocha

**Affiliations:** 1 IAME, UMR 1137, INSERM, Université Paris Diderot, 75018 Paris, France; 2 AP-HP, Laboratoire de Bactériologie, Hôpital Bichat, 75018 Paris, France; 3 Ecole doctorale Frontières du vivant, Université Paris Diderot, 75013 Paris, France; 4 Microbial Evolutionary Genomics, Institut Pasteur, CNRS, UMR3525, Paris, 75015 France; 5 Hub de Bioinformatique et Biostatistique – Département Biologie Computationnelle, Institut Pasteur, USR 3756 CNRS, Paris, France; 6 Genomics Platform, BIOMICS, Institut Pasteur, 25-28 rue Dr Roux, Paris, 75015 France; 7 AP-HP, Laboratoire de Génétique Moléculaire, Hôpital Bichat, Paris, France

**Keywords:** Isolation, separation and purification, Bacteriophages

## Abstract

The microbiota of the human gut is a complex and rich community where bacteria and their viruses, the bacteriophages, are dominant. There are few studies on the phage community and no clear standard for isolating them, sequencing and analysing their genomes. Since this makes comparisons between studies difficult, we aimed at defining an easy, low-cost, and reproducible methodology. We analysed five different techniques to isolate phages from human adult faeces and developed an approach to analyse their genomes in order to quantify contamination and classify phage contigs in terms of taxonomy and lifestyle. We chose the polyethylene glycol concentration method to isolate phages because of its simplicity, low cost, reproducibility, and of the high number and diversity of phage sequences that we obtained. We also tested the reproducibility of this method with multiple displacement amplification (MDA) and showed that MDA severely decreases the phage genetic diversity of the samples and the reproducibility of the method. Lastly, we studied the influence of sequencing depth on the analysis of phage diversity and observed the beginning of a plateau for phage contigs at 20,000,000 reads. This work contributes to the development of methods for the isolation of phages in faeces and for their comparative analysis.

## Introduction

Our understanding of human gut microbiota has drastically increased in the past decade, but the majority of gut metagenomic studies have focused on the bacterial component of the microbiota in healthy subjects and in patients suffering from various pathological conditions. The human gut virome consists of the whole community of viruses in the gut and is mainly composed of bacteriophages (henceforth called phages)^1^. In human gut, there are between 10^9^ to 10^12^ virus-like particles per gram of faeces^2,3^, a density within the order of magnitude of bacteria. The contribution of phages to gut microbiota ecology and their effects on human host are just beginning to be highlighted^4,5^. As an example, an expansion of the number of enteric Caudovirales phages has been observed in patients with Crohn disease and ulcerative colitis^6^.

The human gut phageome varies according to the age of individuals. The infant phageome is highly dynamic and associated with early life changes in its composition [8]. In contrast, the phageome of adults is stable^7^. Healthy adults have a gut phageome mainly constituted of temperate DNA phages belonging to the Caudovirale order (Siphoviridae, Myoviridae, Podoviridae)^3,8^. There is a high inter-individual variation between the phageome of healthy individuals^9^, but a recent study showed that a small set of phages are found in the majority of healthy people^10^. One of these is the crAssphage, a 97 kbp Podoviridae phage that is highly abundant and ubiquitous in the human gut metagenome^11^. Our limited knowledge about gut phages is reflected in the limited host taxonomic span of sequence databases and results in that the vast majority of reads in human viromes cannot be annotated into a functional or taxonomic category^12^.

In the context of a project where we study the bacterial microbiota of the faeces of several healthy individuals during several months^13^, we looked for a simple, efficient, reproducible and inexpensive methodology for (i) phage isolation from faeces, (ii) DNA extraction and sequencing and (iii) computational analyses of the sequencing data. The methods developed for these tasks are very diverse, making comparisons between studies difficult (Table S1). Recently, two studies^14,15^ have compared different methods to isolate phages from faeces. Many of the methods in Castro-Meija *et al*.^14^ have merits, but they are not easily scalable to hundreds of samples because they all include a cesium chloride gradient (CsCl) step, which is complex and time consuming. Five other phage isolation methods were presented in Kleiner *et al*.^15^, including a method using polyethylene glycol (PEG), which is cheaper, faster and more easily scalable, compared to CsCl. However, they failed to use it due to the formation of a viscous mass. After the phage isolation and DNA extraction step, most of the methods found in phageome papers used an amplification step (called multiple displacement amplification, MDA) of phage DNA before sequencing (Table S1). MDA is known to introduce biases associated with sequence composition^16,17^ and depending on the exact method it can produce a large excess of ssDNA phages^18^. This has been well documented for mock communities^19,20^, but not for human gut samples.

The goal of this work was to produce an easy, reproducible and low-cost methodology  to analyse a large number of samples of gut phageome, from viral isolation to bioinformatic analysis. To this purpose, we have tested five different methods of phage isolation. For the one we favoured by the results, we assessed its reproducibility, estimated the bias of an MDA step before sequencing, the effect of sequencing depth, and developed bioinformatic approaches. The prophages of bacterial genomes are ignored in the study, because they are retrieved when sequencing the bacterial fraction of the samples.

## Results

### A scalable, efficient and cheap method for phage isolation from faeces

We started our study by testing five different methods to isolate phage DNA from faecal samples (described in Table 1). These methods differ mainly by the method of concentration of phages in the sample: method I uses an ultrafiltration technique, method II an ultracentrifugation and an ultrafiltration technique, method III an ultracentrifugation step followed by a CsCl ultracentrifugation, method IV a PEG concentration step and method V a simple ultracentrifugation step. In terms of feasibility, methods II, III and V required an ultracentrifuge, unlike methods I and IV that can be set up easily in a routine lab. The yield of the method, in nanograms of DNA per gram of faeces, varied by a factor of 20 (Table 1). The reference method using CsCl (method III), produced small amounts of DNA and required an additional MDA step before sequencing, while other methods did not. We also estimated the prices of the different concentration steps - including only the reagents - and the working time for phage isolation (Table 1). Method IV was, by far, the cheapest method whereas methods I and V were the fastest. Of note, the time of the method IV included an overnight incubation at 4 °C.

**Table 1:** Main characteristics of the analyses performed on the AA_0_ faecal sample.

	AA_0_				
**Phage isolation**					
Method	I	II	III	IV	V
Quantity (gr)	5	5	5	1	1
Filtration (µm)	2 0.45 0.22	2 0.45 0.22	2 0.45 0.22	2 0.45	2 0.45
**Concentration**					
Method	UF	UC UF	UC UC-ClCs	PEG	UC
Price (Euros)/sample	12.5	17.5	23	0.4	5
**Time (WD)**	0.5	1	1.5	1*	0.5
**DNA extraction**	+	+	+	+	+
**DNA quantity** (ng/gr of faeces)	1058	61.5	54	107	77
**MDA**	−	−	+	−	−
**Sequencing**					
Low coverage Number of reads	+“\n”557,844	+“\n”1,052,700	+“\n”618,938	+“\n”1,183,870	+“\n”2,112,614
High coverage Number of reads	−	−	−	+87,148,950	−

Abbreviations: UF-Ultrafiltration, UC-Ultracentrifugation; CsCl-Cesium chloride, PEG- Polyethylene glycol, WD-Working day, MDA- Multiple displacement amplification,

*Including O/N incubation.

To assess the efficiency of the methods, we sequenced the extracted DNA at low coverage (LC) and obtained 5,525,966 reads, *i.e*., an average of 1,105,193 reads per method (details in Table 1). The reads were subjected to quality control and then assembled per method, producing a total of 110,709 contigs. The distribution of the sizes of contigs showed a majority of extremely small contigs (median = 389 bp). We excluded all contigs with less than three open reading frames (ORFs) because these analyses are prone to contamination with small amounts of DNA from bacteria and because very small contigs are hard to identify as phages. This reduced the dataset to 3,369 contigs that represented only 3% of the number of contigs, but were mapped by 84.5% of all reads. Hence, this threshold reduces dramatically the number of contigs, but not the number of reads actually used in the analysis.

**Figure 1:**
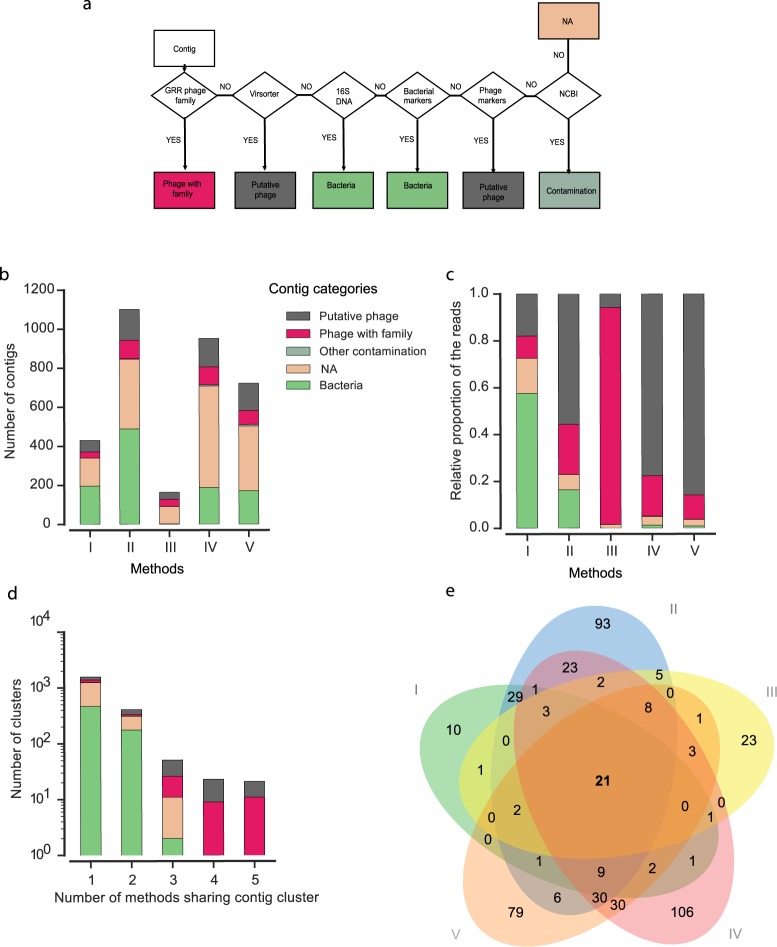
Algorithm to explore contigs and comparison between the five different phage isolation methods (I to V) used to analyse the faeces sample AA_0_ of the volunteer AA. (**a**) Flowchart for the taxonomic attribution of the contigs with a complete method. It permitted to classify a contig according to the following groups: “phage with family”, “putative phage”, “bacteria”, “contamination”, “non-attributable (NA)” according to wGRR strategy, Virsorter software, HMM bacterial markers and 16S profile and NCBI database. (**b**) Number of contigs with a minimum of three ORFs, after a rarefaction step, classified following the procedure in (**a**). (**c**) Frequency of reads mapping the different classes of contigs. (**d**) Number of methods sharing contig clusters. For example, 21 contig clusters (all categorized as phage) were retrieved by all the methods. (**e**) Venn diagram of the number of phage clusters identified for each method (only clusters where the referenced sequence is classified as “phage with family” and “putative phage”). NA: non-attributable.

To compare the samples in terms of richness (the number of contigs and their content), each sample was rarefied at 322,285 reads. The contigs were then classified as bacteria, phage with family, putative phage, other contamination or non-attributable (NA), using the flowchart described in the Methods (see Fig. 1a). The number of contigs, and their categories, were very diverse across the different methods (Fig. 1b). Method III presented the smallest number of contigs, in opposition to methods II and IV. The majority of contigs could not be assigned to phages or bacteria. But when we weighted each contig by the number of reads mapping it (Fig. 1c), we observed that the vast majority of reads maps the contigs classified as phages in all but the first method, which showed high levels of bacteria DNA. All methods produced phage contigs with median size around 5 kb (Fig. S1).

We then compared the similarities between contigs produced by each method. We clustered all contigs by sequence similarity and assessed the number of methods that contributed for each cluster of contigs (for a variant using more local alignment and producing similar results, see Fig. S2). The resulting 2,075 clusters of non-redundant contigs were mostly identified by one or two methods (Fig. 1d). However, the few contigs identified by four or all of the methods were all classed as phages (or putative phages). A Venn diagram (Fig. 1e) describing the phage (or putative) contigs indicates that methods II and IV exhibited the most diverse sets of phage contigs.

These results led us to continue our study using method IV because it is scalable and rapid (excluding the overnight incubation) while producing a large number of very diverse phage contigs, with relatively little contamination and at a low cost.

### The effect of sequencing depth

**Figure 2:**
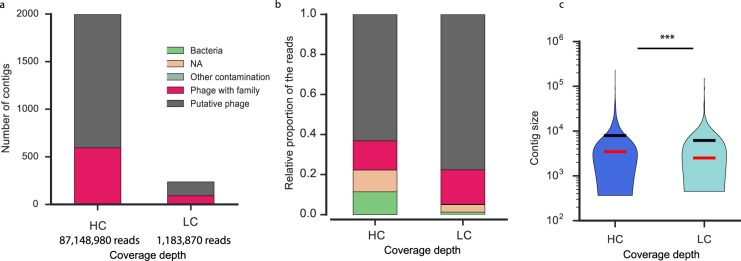
Comparison of the phage contigs obtained from the same sample (AA_0_) with different sequencing depths: low coverage (LC) and high coverage (HC). (**a**) Number of phage contigs (classes “phage with family” and “putative phage”) detected in the two datasets. (**b**) Relative proportion of the reads for the different categories (classified with the flowchart presented in Fig. 1a) in the two datasets. (**c**) Violin plot of the distribution of the sizes of phage contigs in the two datasets. The red line corresponds to the median and the black line to the mean. ***Significant difference, Wilcoxon test, p < 0.001. NA: non-attributable.

The depth of sequencing is often decided based on budget constraints. Nevertheless, it is important to assess how much is lost or gained when making this decision. To tackle this point, we sequenced the same sample extracted with method IV at a high coverage (HC) (87,148,950 reads) (Table 1). Statistics comparing this dataset and the previous one (LC, 74 times fewer reads) are described in supplementary material (Table S3). Each dataset was assembled separately, the LC dataset produced 952 contigs (with at least three ORFs) and the HC dataset produced 16,608 contigs (with at least three ORFs). We searched for phages (phages with family or putative phages) on these two pools of contigs using the developed flowchart (Fig. 1a), and found 237 phage contigs in the LC dataset versus 1994 in the HC dataset (Fig. 2a). The number of phage contigs, weighted by the number of reads mapping on them, was very large in both datasets, even if the HC dataset showed more contaminants or unassigned contigs (Fig. 2b). The distribution of sizes of these contigs was significantly different: phage contigs from the HC dataset were significantly longer (mean size of 7,926 bp *vs* 6,163 bp, P < 0.001, Wilcoxon test, Fig. 2c). Hence, the HC analysis has 79 times more reads, but the average contig is only 28.6% larger.

We pooled phage contigs (phage with family and putative phage) from the two datasets and clustered them using CD-HIT. We found 189 clusters that included contigs from both datasets. We found 1803 phage contigs that were exclusively present in the HC dataset, showing that increasing the depth of sequencing significantly increases the number of novel phage contigs. On the other hand, 41 phage contigs were exclusively present in the LC dataset. To enquire on the possibility that these 41 contigs were only partly homologous to contigs in the HC dataset, leading them not to cluster together, we re-ran the CD-HIT with a local alignment (option –G 0) and with a less strict coverage on the shorter contig of 50% instead of 95%. This resulted in only 14 contigs exclusively present in the LC dataset (Table S4) representing 2% of the LC reads. Hence, very few contigs assembled in the LC dataset are absent from the HC dataset.

**Figure 3:**
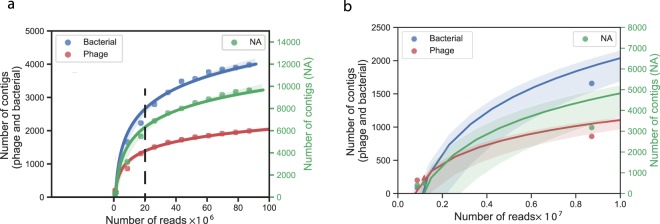
Analysis of the effect of sequencing depth on the number and type of contigs. (**a**) Contigs obtained by re-sampling the high coverage (HC) dataset at different depths. The black y-axis on the left indicates the number of contigs of phage and bacteria. The green y-axis on the right indicates the number of “NA” contigs. The vertical black dashed line represents the sequencing depth recommended (20,000,000 reads). (**b**) Zoom of (**a**) on 0 to 10 millions of reads, simulated data are in circles and observed data [from the low coverage (LC) dataset] in triangles. NA: non-attributable.

We quantified the gains associated with increased sequencing by simulating different sequencing depths. This was done by randomly selecting reads (in-house script) from the sample IV (HC sequencing). We performed steps from 1% to 90% in steps of 10 (Table S5). Each subset was assembled and analysed independently (in the same way as before). To test the reliability of this simulation method, we plotted on the same figure the number of phage, bacteria and NA contigs for simulated datasets and for the LC dataset. The number of contigs identified in the LC dataset is very close to the curve fitting the results of the simulations from the HC dataset (Fig. 3b), confirming the adequacy of our simulations. Expectedly, the number of contigs increases less than linearly with the number of reads: smaller datasets have proportionally more contigs. Interestingly, the abundance of phage contigs, relative to bacterial and unassigned contigs, decreases as the sequence depth increases. This suggests a lower diversity of phage DNA, relative to other elements of the community, and faster saturation of this population.

This analysis does not reveal any obvious point where to stop sequencing a sample, because increasing sequencing depth always provides more information. We observed a decreasing number of novel contigs with increasing sequencing depth. The number of novel phage contigs initiate a plateau after 20,000,000 reads (Fig. 3a), from this point the vast majority of novel contigs are not classed as phages.

### MDA decreases the reproducibility of the isolation method

To assess the reproducibility of method IV and how it is affected by MDA, we extracted phage DNA from faeces of three healthy volunteers (AA, BB, CC) (Table 2). Each faecal sample was separated in nine samples: one set of three replicates was treated as above (S), another set of three replicates was amplified by MDA for a short period (SP30), and a final set of three replicates was amplified for a longer period (SP90). The sequencing of these samples resulted in 22,241,488 reads, for an average of 823,758 reads per sample. All reads from the 27 samples were assembled together to obtain a contig reference catalog called ABC-Reference-Contig-Catalog that contained 6,056 contigs with a mean size of 4,796 bp and a median of 2,173 bp. We then mapped the reads of each sample on this set of contigs. The resulting count matrix was rarefied at 716,808 reads to allow straightforward comparisons across samples.

**Table 2:** Main characteristics of the analyses performed on the faeces of the three human volunteers (AA, BB, CC) for assessing the reproducibility of the method IV and the effects of MDA.

Samples																											
Samples	AA^a^									BB									CC								
Replicates	1			2			3			1			2			3			1			2			3		
Method	IV			IV			IV			IV			IV			IV			IV			IV			IV		
Sephadex	YES			YES			YES			YES			YES			YES			YES			YES			YES		
**For each replicate, 3 different amplification methods were tested**																											
No MDA	✔			✔			✔			✔			✔			✔			✔			✔			✔		
MDA 30′		✔			✔			✔			✔			✔			✔			✔			✔			✔	
MDA 90′			✔			✔			✔			✔			✔			✔			✔			✔			✔
Sequencing	✔	✔	✔	✔	✔	✔	✔	✔	✔	✔	✔	✔	✔	✔	✔	✔	✔	✔	✔	✔	✔	✔	✔	✔	✔	✔	✔

^a^The AA and AA_0_ (see in Table 1) faecal samples correspond to two different faecal samples from the same individual.

Abbreviations: MDA-Multiple displacement amplification.

**Figure 4:**
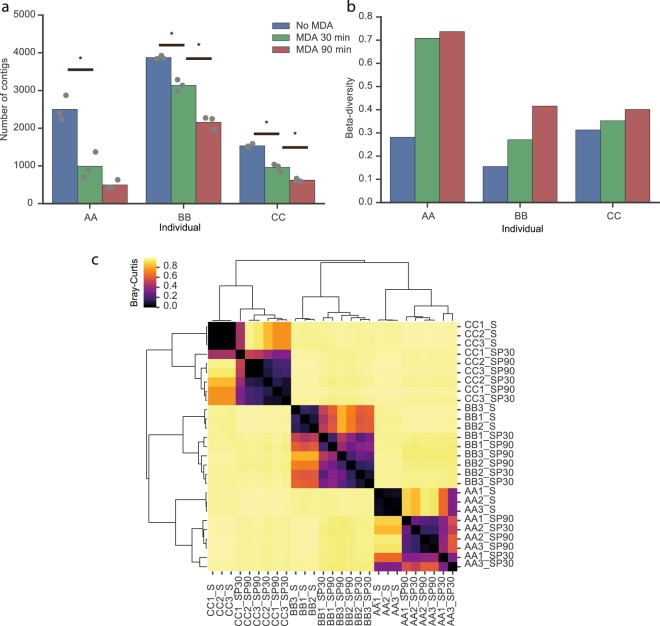
Analysis of the diversity and reproducibility of phage extraction method IV on the samples of three individuals (AA, BB, CC) using two protocols of multiple displacement amplification (MDA) (No MDA, 30 minutes MDA, 90 minutes MDA). (**a**) Number of different contigs (alpha-diversity) in the 27 samples. The bars indicate the average values. The differences in the values of pairwise alpha-diversity between S, SP30 and SP90 are statistically different (*P < 0.05, t-student test). (**b**) Beta-diversity computed between replicates. (**c**) Heatmap of the Bray-Curtis dissimilarity between the 27 samples. The trees above and on the left of the heat map were obtained using hierarchical clustering with the function cluster.hierarchy.linkage (method ward) from the library SciPy (v1.0.1) of python.

MDA resulted in the fewer different contigs, an effect that increased with the amplification period of time (P < 0.05, student t-test). This conclusion remains significant when we adjusted the number of contigs to account for their average size. This shows that MDA decreases the diversity of contigs observed in the samples. The composition of the samples from the same faeces was expected to be more similar (and thus show lower beta-diversity) than that of samples from different faeces. We thus computed the beta-diversity between replicates to assess the reproducibility of the methods. The method without MDA presented the lowest beta-diversity in each healthy volunteer, and longer periods of MDA lead to increased values of beta-diversity (Fig. 4b). We computed a heatmap of the Bray-Curtis dissimilarity between all the 27 samples, complemented with hierarchical clustering to assess the similarities between samples (Fig. 4c). This analysis showed a very clear clustering of the samples by individual. Yet, some amplified samples had very high within-individual Bray Curtis dissimilarity, and SP30 and SP90 samples were sometimes intermingled in the hierarchical classification. Overall, the tightest and most consistent clusters were always those of non-amplified samples, which showed an average intra-group Bray Curtis dissimilarity much smaller than the others (Fig. S3). These samples always clustered together in each individual, contrary to those of the other two methods. In summary, MDA severely decreased the genetic diversity and the reproducibility of the method, and method IV without MDA showed high reproducibility and ability to discriminate between individuals.

### The wGRR strategy informs the phageome

We developed a method to attribute a family, a lifestyle or a host to contigs. We searched for sequence similarity between the contigs and (pro)phages of three databases (phage Refseq database, prophages from HMP bacteria and prophages from RefSeq bacteria, see Methods). These results were used to compute a score of similarity called weighted gene repertoire relatedness (wGRR) for each pair of contig/phage. This corresponds to the assignation of the properties of the best hit (phage, prophage) to the global characteristics of the contig (family/host/lifestyle attribution), which facilitates processes that aim at classing the entire contig. To quantify the quality of such assignments, we defined a wGRR cut-off per database. We computed the wGRR of each pair of phages of the Refseq database and found that a wGRR cut off of 5.2 gives 95% correct predictions of phage family (Fig. S4a). However, this cut-off is valid only for comparisons between complete phages. We produced sets of fragments of phage genomes of different sizes to simulate phage contigs of different size. We then recalculated the wGRR cut-off between the complete phage sequences and these different sets (Fig. S4b,c). This produced a smooth increase of the wGRR threshold value with decreasing contig size that could be fit with a negative exponential function (Fig. S4d). We used a similar approach to define wGRR thresholds for lifestyle and host attributions (Table S6).

**Figure 5:**
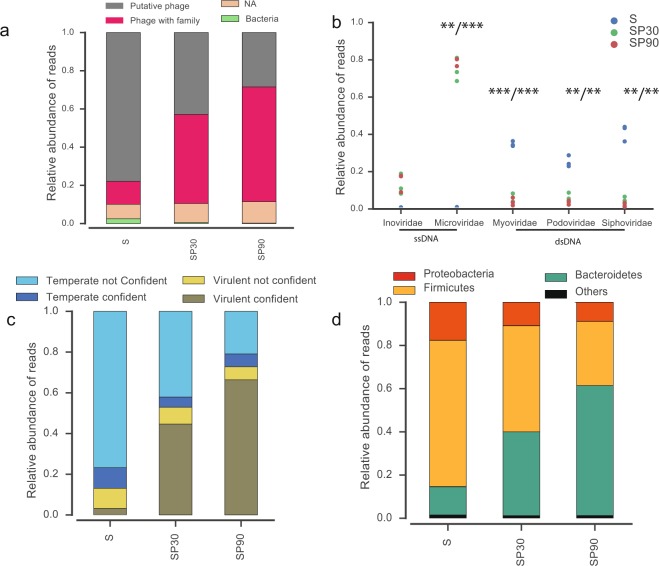
Analysis of the contigs of the healthy volunteers’ (AA, BB, CC) samples. (**a**) Bar plots of the relative abundance of reads for each contigs category in each method: method IV for phage isolation following with a 30 min multiple displacement amplification MDA (SP30), 90 min MDA (SP90) or no MDA (S). (**b**) Plot of the relative abundance of reads for each phage family. For Microviridae, Myoviridae and Siphoviridae the relative abundance of reads are significantly different between MDA protocoles (SP30, SP90) and no MDA protocoles (S), as assessed by paired t-tests (* < 0.05, ** < 0.01, *** < 0.001). (**c**) Bar plots of the relative abundance of the reads mapping the contigs of a certain lifestyle. (**d**) Bar plots of the relative abundance of the reads mapping the contigs of a certain host phyla. NA: non-attributable.

We then used our method to characterize the phage contigs in terms of family, host and lifestyle attribution and assess the differences between samples in terms of their level of MDA (using the ABC-Reference-Contig-Catalog, details in Table S7). The vast majority of reads were classed as phage contigs. MDA had little effect on the amount of non-assigned or bacteria contigs (Fig. 5a). The ability to identify their family increased with the period of MDA (Fig. 5b). The method without MDA (S) showed a large majority of double stranded DNA (dsDNA) Caudovirales (Siphoviridae, Myoviridae, Podoviridae) and very few single stranded DNA (ssDNA) phages (Inoviridae, Microvirdiae). This is expected on two accounts. First, dsDNA phages are thought to be the most abundant^3^. Second, ssDNA phages are not sequenced using the Nextera XT DNA Library Prep Kit that we have used. The use of MDA (SP30 and SP90) drastically increased the proportion of ssDNA phages (Inoviridae, Microviridae) and almost excluded dsDNA phages (Fig. 5b). The predicted lifestyle of the contigs was also very different between methods, with a dramatic increase in contigs classed as virulent phages in samples with MDA; a consequence of the fact that most (73%) ssDNA phages of phage Refseq database are classed as virulent in PHACTS^21^ (Fig. 5c). MDA was also accompanied by an increase of Bacteroidetes phages (Fig. 5d), in agreement with works identifying many Microviridae in this clade^2^.

### Biological insights

Our analyses revealed the following categories in the ABC-Reference-Contig-Catalog: 57.6% of the contigs could not be assigned, 18.5% were classed as putative phages, 15% as bacteria and 8.3% to a known family of phages. Importantly, the order of decisions in the decision algorithm used in Fig. 1a has little impact on the overall conclusions since very few contigs are ever classed as phage and bacterial or phage and contamination (all details are in Fig. S5). Intriguingly, 218 out of 506 contigs classified as “Phage with family” were not detected by Virsorter software (Fig. S5). These contigs are small and this is probably the reason why they were missed by Virsorter (Fig. S6a) even if they have homologs of phage genes. To check that these 218 contigs provide reliable information, we re-analysed Fig. 5 (panels b–d) after removing the contigs missed by Virsorter. The results are similar (Fig. S6b–d), showing that the wGRR approach provides a means of classifying the phage contigs and of identifying novel ones.

We found that only 0.41% of the contigs corresponded to other types of contamination: 2 contigs matched eukaryota DNA, 1 contig matched archaea DNA and 22 contigs had sequence similarities to environmental DNA from uncultured bacteria found by metagenomic methods. Most contigs did not match any database but when we looked at the relative abundance of reads mapped on ABC-Reference-Contig-Catalog, we observed that in method S the vast majority of reads mapped on putative phage or phage contigs (Fig. 5a). We studied more precisely the composition of the gut phageome of the three volunteers when the procedure did not include MDA (Fig. S7). Here, we observed that Caudovirales were largely predominant, but within this group each volunteer had a different predominant family (Podoviridae for AA, Myoviridae for BB and Siphoviridae for CC).

The analysis of the phage lifestyle showed that nearly half of the phage contigs could not be classified according to the lifestyle (46.6%) (Table S7B). The vast majority of the remaining contigs were classed as temperate phages in the non-amplified dataset, although most of the times as “non-confident”. Lastly, most predicted hosts of the gut phages correspond to bacterial phyla frequent in the gut (Firmicutes, Bacteroidetes, and Proteobacteria)^22,23^ (Table S7C).

The crAssphage is a 97 kbp bacteriophage that is frequently found at high abundance in human faeces^11^. It infects *Bacteroides intestinalis*^24^. We could not identify the originally identified crAssphage when we mapped (using bowtie2) our reads on the phage DNA sequence. But when we analysed homologies between proteins encoded in the contigs and the proteins of the phage, we found three contigs with many distant homologs to proteins of the crAssphage (with respectively a wGRR of 19, 27 and 26, Fig. S8). All these contigs were found in all the replicates of the three healthy volunteers with or without MDA, even if the amplification decreased its frequency, as expected since it’s a dsDNA phage^25^. Overall, this suggests that our samples include phages distantly related to the crAssphage.

## Discussion

There is still a need to standardize protocols to isolate, sequence, and analyse phages from faeces. Here, we tested different phage isolation methods on a single faeces sample to identify a method that could be used in projects with many samples. We decided to test our method (PEG) in real samples, using multiple replicates and different sequencing depths. This allows assessing how it fares in complex samples, at the cost of not knowing the accuracy of the method because its exact composition is unknown. The use of mock communities in the future could allow to quantify the association between the coverage of the contigs and their abundance in the sample, even if the poor current knowledge of most of the human gut phages makes such analyses hard to translate into the context of natural complex communities. Accordingly, the majority of contigs in our samples have no homology to known phages (or bacteria).

All the methods had a centrifugation step that was followed by the filtration of the supernatant; this was performed to eliminate debris. Bacteria were subsequently eliminated by a filtration step and each specific step of phage concentration were performed. We observed a high variability in terms of contigs provided by the five methods, which highlights the difficulty of comparing phageomes obtained with different procedures. The small size of contigs is quite typical of metagenomics datasets. The extraction techniques may lead to damage and fragmentation of the DNA and contribute to the existence of so many small contigs. If so, the effect is similar for the five extraction techniques because we find small contigs in all the samples. The historical reference method, Cesium chloride phage isolation, gives the lowest bacterial contamination and the largest contigs. However, it is very fastidious, has the lowest phage diversity, and shows poor reproducibility, in agreement with a previous study^15^. This may be due to the need to amplify DNA given the low DNA yield of the method when dealing with small samples. In any case, the cost and time required for this method make it poorly applicable to large-scale studies. Other isolation phage methods tested in this study provided interesting results. For technical reasons, we could only analyse one sample per method. Hence, we cannot claim that the method we have chosen is necessarily better than the other methods. However, the PEG isolation method presented good reproducibility and the best cost/efficiency ratio, which are very important for large-scale studies. Besides, it produced a large number and a high diversity of phage contigs.

Some steps performed in the method IV (PEG) may have contributed to its good performance: (i) the initial mechanic agitation could help to better dissolve phage faeces in PBS (ii) filtration step with 2 µm and 0.45 µm filters, and not 0.22 µm filter as in method I to III. It has been shown that the 0.22 µm filter reduced by half the number of viral particles observed under the microscope^3^ (iii) the PEG concentration method seems to be an efficient method to concentrate phages.

For many years, due to the low amount of phage DNA extracted and the high amount of DNA required in the library preparation kits, research teams had to use MDA before sequencing. This study shows, along with several others^16,17^, that MDA amplifies preferentially ssDNA. There is some controversy on the relative frequency of dsDNA Caudovirales or ssDNA microviridae in the healthy human gut^7–9,26^. Some of these works used a MDA step before sequencing, which preferentially amplifies ssDNA^6–9^, whereas others - like ours - used library preparation kits that don’t account for ssDNA phages^19^. Microscopy studies^3^ of gut phageome showed a majority of dsDNA phage (from the Caudovirales family). Recently, the use of a novel commercial kit allowed preparing dsDNA and ssDNA libraries compatible with Illumina sequencing in six aquatic samples, and revealed the frequent presence of ssDNA viruses. Nevertheless, these were vastly outnumbered by dsDNA viruses in all cases^19^. It remains to be tested in gut phageome. Independently of the real frequency of ssDNA phages in the gut, MDA leads to less diverse and reproducible phage contigs, posing a problem for the analysis and comparison of multiple samples. Hence, MDA should be avoided, at least when studying the dsDNA component of this community.

Phages evolve rapidly by exchanging functional modules by recombination^27^, and genome databases do not adequately represent the diversity of the phage world. As a consequence, phage genomes have many genes lacking clear homology to known genomes. To analyse the gut phageome, a global genomic similarity approach can provide interesting additional information relative to approaches based on local similarity. This spurred our approach of assembling reads into contigs and then search similarity to known phages using a global approach (the wGRR index). This index has been previously used to analyse several types of mobile genetic elements, including phages^28,29^. Here, it allowed the inference of the family, lifestyle and host phyla of many phage contigs, representing a large fraction of the reads. This method is complementary to typical individual protein-based sequence similarity analyses since the wGRR index collects information on the presence of homology and degree of identity of the set of genes of the contig. This global index is thus particularly appropriate to make classifications of the entire contigs. Its limit is that if one wishes to change the reference database, then the fit of a new wGRR cut-off equation will be needed. For example, since the beginning of our work, several novel programs were published to identify prophages in bacterial genomes with higher sensitivity or specificity than PhageFinder. We made a rapid analysis of the effect on the wGRR method of changing the source of prophages by using Phaster^30^ instead of PhageFinder. It showed that the introduction of a more accurate method further improves the analysis of wGRR, since the threshold upon which classifications can be made decreases (Fig. S9). The wGRR method is general and can be used with any prophage finder method.

The majority of the reads present in samples mapped phage or putative phage contigs, supporting the quality of our isolation method. Nevertheless, the majority of the contigs could not be classed. Most of them are small and poorly covered, they account for a small amount of reads (Fig. S1), which may explain the lack of homologous sequences in the databases. Finally, some contigs were classed as of bacterial origin. It is not possible to determine the relative frequency of them corresponding to bacterial contamination or to virions carrying bacterial DNA arising from generalized transduction.

The phageome of each individual is unique^7^. This study shows that Bray-Curtis dissimilarity between two gut phageomes content is much smaller for samples from the same individual than for samples from different individuals. The Bray-Curtis dissimilarity between two replicates is low (0.08). This provides a measure of the error of the method, and suggests that the method is reproducible.

## Conclusion

The phages of the gut have a lot to teach us, so it is essential to know how to isolate them and to extract and analyse their genomes. Our study contributes to the standardization of the methods used to study faeces phageome. It should provide reliable comparisons between studies from different laboratories. Future studies could aim at combining on the same sample the phageome and microbiome analysis to better characterise microbial communities.

## Methods

### Sample collection

A faecal sample (AA_0_) from a healthy adult volunteer (AA) was used to compare the five phage isolation methods designated I to V (Table 1). To compare the reproducibility of method IV and to analyse the bias of MDA procedure, three unrelated healthy adult volunteers (AA, BB and CC) were recruited to provide one faecal sample (Table 2). Samples were stored at 4 °C (for a maximal period of 24 h), homogenized with a Stomacher paddle blender (Interscience, France), and stored at −80 °C.

### Phage isolation and DNA extraction

All isolation methods are summarized in Table 1. In each of methods I, II and III, 5 gr from AA_0_ were weighed and homogenized in 40 ml of phosphate buffered saline (PBS) (Sigma-Aldrich). Samples were centrifuged three times at 3,000 g for 20 min by taking the supernatant at each time. Then, the supernatant was filtered successively with a 2 µm (hydrophilic glass fibre, Millipore), 0.45 µm (Polyethersulfone membrane, Millipore) and 0.22 µm (Polyethersulfone membrane, Millipore) membranes.

Various procedures were then performed to enrich for phages.

- In method I, a double ultrafiltration was performed on the filtrate with an Amicon Ultra-15 (Merck Millipore) during 20 min at 5,000 g in a swinging out rotor as described in^7^.
- In method II, the filtrate was ultracentrifuged at 100,000 g during 2.5 hours at 4 °C, the phage pellet was suspended in 10 ml of Tris-HCL, NaCl (TN) buffer and then ultrafiltrated with Amicon Ultra-15 (Merck Millipore) during 20 min at 5,000 g in a fixed rotor as described in^8^.
- In method III, the filtrate was ultracentrifuged at 100,000 g during 2.5 hours at 4 °C and the phage pellet was suspended in 6 ml of TN buffer. The sample was then loaded onto a CsCl gradient composed of 1.7 g/ml, 1.5 g/ml, 1.35 g/ml steps and centrifuged for 2.5 hours in a SW Ti-41 rotor at 4 °C. The interface between the 1.35 and 1.5 g/ml region was removed based on the protocom published by Thurber *et al*.^31^ and CsCl was removed by dialysis.

In each of methods IV and V, 1 gr from AA_0_ was weighted and homogenized in 40 mL of PBS.

- In method IV, the sample was then agitated with a mechanic laboratory agitator during 1 hour at 4 °C at 900 rpm (VWR incubating mini shaker), centrifuged at 17,000 g for 5 min and the supernatant was filtered at 2 µm and 0.45 µm. Phages were then concentrated using a PEG method. One molar solid NaCl and 10% (v/v) PEG 8000 (Sigma) were dissolved into the filtered culture fluid and incubated overnight at 4 °C as recommended for a constant and stable precipitation^32^. The solutions with the phages were pelleted by centrifugation at 5,250 g for 1 h at 4 °C and re-suspended in 500 µL of SM buffer (NaCl 100 mM, MgSO4.7H_2_O 8 mM, Tris-Cl 50 mM).
- In method V, the sample was centrifuged twice (one at 2,500 g for 5 min and one at 5,000 g for 15 min), and the supernatant was filtered at 2 µm and 0.45 µm. The filtered fluid was ultracentrifuged at 100,000 g during 2.5 hours at 4 °C in a SWTi-41 rotor as described in^15^. The phage pellet was then suspended in 1 mL of SM buffer.

All samples were treated with 10 U/ml of DNAse (Sigma) for 30 min at 37 °C followed by 10 min at 65 °C to stop the reaction. DNA was then extracted using the commercial kit “Phage DNA extraction” (Norgen biotek Corp, Ontario, Canada). DNA extracted from method III was amplified with phi-29 DNA polymerase (REPLI-g Mini kit, Qiagen, Germany) (MDA step) as recommended by the manufacturer because there wasn’t enough DNA to prepare the sequencing library.

To test the reproducibility of method IV and assess the potential bias of MDA, we produced three different faecal samples (from three different individuals AA, BB and CC) and isolated phages on three different aliquots from each faecal sample (Table 2, 27 samples in total). An additional DNA purification step with a Sephadex column (Sigma-Aldrich, France) was done for the 27 samples in order to improve the purity of DNA. To assess the bias of MDA we made a replicate without MDA before sequencing (S), another with a 30 min MDA (SP30) and the last one with a 90 min MDA as recommended by the manufacturer (SP90) (Table 2).

### DNA sequencing

Phage DNA was sequenced with the Illumina MiSeq Nano V2 PE_250 bases method using the Kit Nextera XT with an input of 1 ng DNA, called low coverage (LC) sequencing. Phage DNA from method IV was also sequenced with a deeper coverage with Illumina Hiseq2500 PE_250 bases, called high coverage (HC) sequencing. All samples were sequenced using paired-end reads (numbers of reads mentioned in this manuscript always refer to paired-end reads).

### Genome sequences

The sequences and corresponding annotations of 1,943 complete bacteriophage genomes were retrieved from GenBank Refseq (ftp://ftp.ncbi.nih.gov/genomes; last accessed November 2016) called phage Refseq database in this paper. The order, the family, the type of nucleic acids and the bacterial host were also extracted from the GenBank files of each phage (when available). This dataset contained 1,756 Caudovirales (90%) of which 3 main families were identified, i.e.: 53% were Siphoviridae, 27% Myoviridae, and 19% Podoviridae. In order to create prophage databases we retrieved bacterial genomes from different locations: (i) 5562 complete bacterial genomes from Refseq GenBank (ftp://ftp.ncbi.nih.gov/genomes/, last accessed in November 2016) and (ii) 457 draft genomes of the Human Microbiome Project (HMP) (http://hmpdacc.org/HMRGD, last accessed in December 2015).

### wGRR

The weighted gene repertoire relatedness (wGRR) is a similarity score between two genetic elements. We used blastp v.2.6.0 (default parameters) to compute sequence identity between proteins encoded in pairs of contigs (A and B). We kept all bi-directional best hits with an e-value lower than 10^−5^. The wGRR score is computed as:

$$wGRR=\frac{{\sum }_{i}^{p}id({A}_{i},{B}_{i})}{{\rm{\min }}(A,B)}\times 100$$

where A_i_ and B_i_ is the pair *i* of homologous proteins present in *A* and *B*, id(*A*_*i*_,*B*_*i*_) is the percent sequence identity of their alignment, and min(*A*,*B*) is the number of proteins of the contig encoding the fewest proteins (*A* or *B*). The wGRR varies between zero and one hundred; it represents the fraction of homologs in the smallest of the two contigs weighted by their sequence similarity^28^.

### Prophage identification

We identified 9927 prophages in the chromosomes of complete bacterial genomes from GenBank Refseq using Phage Finder v4.6^33^ as in^34^. Many of these prophages were very similar. We clustered them with a wGRR cut off of 95% and we took a representative of each cluster (6426 phages). We used Virsorter v.1.0.3^35^ to identify prophages in draft genomes because this software searches for phages in draft genomes and metagenomic datasets (contrary to Phage Finder). We excluded the putative prophages classed under categories 3 and 6 of VirSorter, which often correspond to other mobile genetic elements (e.g. pathogenicity islands). We thus identified 1243 putative prophages in the draft genomes of the Human Microbiome Project (HMP) (http://hmpdacc.org/HMRGD, last accessed in December 2015).

### Prediction of phage lifestyles

The lifestyle of phages from phage Refseq database was predicted using PHACTS v.0.3^21^. Predictions were considered as confident only if the average probability score of the predicted lifestyle was two standard deviations (SD) away from the average probability score of the other lifestyle, as recommended by the authors (who claim a precision rate of 99% with this parameter). Using these criteria, we classified 54% of phage Refseq database into 420 virulent and 629 temperate phages.

### Trimming, assembly, and mapping of reads

The sequence reads were trimmed (i) to remove the Illumina adapters using Adapter Trimming with cutadapt (v1.12)^36^ with default parameter values (ii) and reads with low quality were removed using UrQt (v1.0.18)^37^ with parameters:–min_QC_length 80–min_read_size 100–min_QC_phred 20. In order to optimize the assembly step, we reduced the redundancy of the reads with the Khmer software (v2.0)^38^, using a k-mer size of 20 and a median k-mer coverage level of 20, which makes a digital normalization and a trimming of sequences based on the k-mer abundance. The resulting reads were assembled using SPAdes 3.10.1^39^ with –meta option. Gene prediction was done using Prodigal (v2.6.2)^40^ with –p meta option. We excluded genes lacking start and stop codons. In order to focus our analysis on contigs sufficiently large to study genetic contexts, we excluded contigs with less than 3 open reading frames (ORFs).

We used CD-HIT (v4.7, option -g 1, -c 0.95, -aS 0.9 or option –g 1 –G 0 –c 0.95 –aS 0.5 for local alignment)^41^ to define families of contigs. For each resulting family, we took a representative contig (the largest) to create a non-redundant contig catalog. We then mapped the samples’ reads on this “contigs reference catalog” using bowtie2 (v2.2.9–local–sensitive-local options)^42^ and exploit SAM files with samtools (v1.3.1 with the following commands: views, sort, index, idxstat)^43^. As result, we obtained a matrix representing the number of reads of a sample (columns) mapping each contig reference catalog (rows) in the dataset.

### Distance and diversity analysis

The count matrix was normalized with the following “total-count” method. We first compute the sum of the number of reads for all contigs (k lines) for a given sample (column S_i_) where *a*_*i,j*_ is the observed count of contig j ∈ {1,…, k} in the sample i ∈ {1, … N}:

$${S}_{i}=\sum _{j=1}^{k}{a}_{i,j}$$

The mean (M) of the column’s sum (S_i_) where N is the number of sample was calculated as:

$$M=\frac{{\sum }_{i=1}^{N}{S}_{i}}{N}$$

Finally, a size factor (SF_i_) was determined for each column (i):

$$S{F}_{i}=\frac{{S}_{i}}{M}$$

Each value of the count matrix was then divided by the size factor of the respective column in order to normalize each column.

We computed the pairwise dissimilarity between the samples using the Bray-Curtis compositional dissimilarity measure between columns of the normalized count matrix. This was done with the skbio python package (v4.2) (http://scikit-bio.org/). For diversity analysis, we rarefied the data, *i.e*., we generated a count matrix where each column had the same sum of reads by randomly sampling the original data. This procedure can result in a waste of significant data, and alternatives have been designed to deal with this problem^44^. Yet, in this work samples were of very similar sizes (by design), so rarefaction can be done without much loss of information. The count matrix was rarefied with the”rarefy” function of the vegan package in R^45^.

The richness of a sample was defined as the number of different contigs (with at least three ORFs). Alpha-diversity was defined as the average of the richness of different samples for a given condition.

Beta-diversity within a set of samples was calculated as:

$${Beta}-{diversity}={\rm{1}}-\frac{{Gamma}-{diversity}}{({Alpha}-{diversity})}$$

where gamma-diversity is the total number of contigs present in the samples (with a minimum of one read that mapped on the contig).

### Identification of phage contigs

Putative phage contigs were detected with Virsorter v1.0.3 (option db2 and –virome 1). Given the small size of contigs, we used putative prophages from all categories given by the software. Additionally, we searched for genes that could constitute markers of the presence of phages. HMMER (v3.0, option–cut_ga)^46^ was performed on proteins with HMM phage profile from PFAM (30.0) and TIGRFAM (15.0) with a keyword like “capsid, portal, protease, replication, tails” (details of the profiles are given in supplementary data, Table S2).

### Identification of bacteria contigs and non-bacterial contamination contigs

To identify bacterial DNA, we searched for the presence of specific bacterial markers in the contigs. We used 128 out of 139 HMM profiles (Table S2) of bacterial conserved marker genes^47^ using HMMER (v3.0, option–cut_ga). We removed 11 profiles from the original dataset because they were matched by some proteins in the phage Refseq database (indicating that they are not specific to bacteria) (Table S2). We also searched the contigs for similarity with a 16 S HMM profile using HMMER (v3.0, option -E 0.1).

To identify contigs representing non-bacterial contamination (human, archaea and eukaryotic virus DNA), we used Blastn (v2.6.0, options -evalue 0.00001 -max_target_seqs. 10 -task megablast, select only match with a coverage higher than 90%) to search for homologs of the contig genes in the NCBI-nt catalogue (last accessed September 2017). We classed the contigs with hits using the NCBI taxonomy, attributed using the python package taxabd (https://github.com/HadrienG/taxadb).

### Classification of phage contigs

We computed the wGRR between all contigs with more than three ORFs and between them and phages from the three phage databases (see above). The contigs were sorted with the following data: phage family (*i.e*., Myoviridae, Podoviridae, Siphoviridae, Inoviridae, Microviridae), lifestyle (virulent or temperate), and bacterial host (phyla) associated with the phage with highest wGRR value. However, since these values were sometimes very low, we defined a statistically meaningful wGRR threshold for each type of information. First, we computed all wGRR values between all pairs of phages (or prophages) in each database. We calculated the wGRR cut-off between two elements of known classification so that if one of the elements had a given classification, the other had at least a probability of 95% of having the same classification. This method provides wGRR thresholds to class phages given the classification of the best hit (*i.e*., given the known phage with highest wGRR).

To apply the previous method to metagenomics, we had to devise a way of dealing with phage sequence fragmentation in small contigs. We recalculated the wGRR cut-off in the same way, except that we made the analyses on fragments of known phage genomes. We used fragments of different sizes (5, 10, 15, 20, 30, 40, 50, and 60 genes). This allowed adjusting the wGRR cut-off to the size of the smallest element (the contig in the real analysis). For each database and for each type of information (family, lifestyle, host) we fitted a negative exponential curve in the data using curve fit function from Scipy package (v1.0.1). This equation has the form:

$$Y=\alpha \times {e}^{-\beta X}+\gamma $$

where Y is the wGRR cut-off and X is the number of proteins encoded in the fragment of the phage, α, β and γ depends of the database used and the information searched.

We then computed the best wGRR between the contig and known phages from the phage Refseq database. The contig was classed using the information of the best hit among the phages if the wGRR is higher that the cut-off determined with the equation above.

We used the wGRR values to classify the phage contigs in terms of taxonomy. The classification of the lifestyle depended on the number of proteins encoded on the contig. We used the PHACTS software prediction for contigs with more than 19 proteins (4 categories: virulent/temperate confident or virulent/temperate non-confident). For shorter contigs, PHACTS provided unreliable classifications, and we used the wGRR approach. For host phyla attribution, we used the wGRR calculated in comparisons with three different phage/prophage databases (see section genome sequences and prophage identification). We only required one hit above the threshold in one database to make a phylum attribution, but we left without classification the contigs for which there was a contradiction between the databases.

### Decision algorithm

Flowchart in Fig. 1a presents the algorithm for taxonomic attribution of the contigs. This permitted to classify a contig according to the following groups: “phage with family”, “putative phage”, “bacteria”, “contamination”, “non-attributable (NA)” according to wGRR strategy, Virsorter software, HMM bacterial markers and 16S profile and NCBI database as described above.

### Ethics approval and consent to participate

This work was approved by the Comité d’Evaluation de l’Ethique des projets de Recherche Biomédicale (CEERB) Paris Nord” (Institutional Review Board -IRB 00006477- of HUPNVS, Paris 7 University, AP-HP). We confirm that all methods were performed in accordance with relevant guidelines and regulations, and that informed consent was obtained from all participants.

## Supplementary information

Table S2

Table S4

Supplementary informations

## Data Availability

DNA sequences are available at the EBI ENA under accession number PRJEB30796. Scripts used for the analyses and the figures can be provided here https://gitlab.pasteur.fr/cdhumier/phage_isolation_to_bioinformatic_approach.

## References

[1] Breitbart M (2003). Metagenomic Analyses of an Uncultured Viral Community from Human Feces. J. Bacteriol..

[2] Kim M-S, Park E-J, Roh SW, Bae J-W (2011). Diversity and Abundance of Single-Stranded DNA Viruses in Human Feces▿. Appl. Environ. Microbiol..

[3] Hoyles L (2014). Characterization of virus-like particles associated with the human faecal and caecal microbiota. Res. Microbiol..

[4] Reyes A, Wu M, McNulty NP, Rohwer FL, Gordon JI (2013). Gnotobiotic mouse model of phage-bacterial host dynamics in the human gut. Proc. Natl. Acad. Sci. USA.

[5] Lengeling A, Mahajan A, Gally DL (2013). Bacteriophages as pathogens and immune modulators?. mBio.

[6] Norman JM (2015). Disease-Specific Alterations in the Enteric Virome in Inflammatory Bowel Disease. Cell.

[7] Minot S (2013). Rapid evolution of the human gut virome. Proc. Natl. Acad. Sci..

[8] Minot S (2011). The human gut virome: Inter-individual variation and dynamic response to diet. Genome Res..

[9] Reyes A (2010). Viruses in the faecal microbiota of monozygotic twins and their mothers. Nature.

[10] Manrique P (2016). Healthy human gut phageome. Proc. Natl. Acad. Sci. USA.

[11] Dutilh BE (2014). A highly abundant bacteriophage discovered in the unknown sequences of human faecal metagenomes. Nat. Commun..

[12] Aggarwala V, Liang G, Bushman FD (2017). Viral communities of the human gut: metagenomic analysis of composition and dynamics. Mob. DNA.

[13] Burdet, C. *et al*. Ceftriaxone and Cefotaxime Have Similar Effects on the Intestinal Microbiota in Human Volunteers Treated by Standard-Dose Regimens. Antimicrob. *Agents Chemother*. **63**, (2019). 10.1128/AAC.02244-18 PMC6535507 30936104

[14] Castro-Mejía JL (2015). Optimizing protocols for extraction of bacteriophages prior to metagenomic analyses of phage communities in the human gut. Microbiome.

[15] Kleiner, M., Hooper, L. V. & Duerkop, B. A. Evaluation of methods to purify virus-like particles for metagenomic sequencing of intestinal viromes. *BMC Genomics* **16** (2015). 10.1186/s12864-014-1207-4 PMC4308010 25608871

[16] Yilmaz S, Allgaier M, Hugenholtz P (2010). Multiple displacement amplification compromises quantitative analysis of metagenomes. Nat. Methods.

[17] Džunková M (2014). Direct squencing from the minimal number of DNA molecules needed to fill a 454 picotiterplate. PloS One.

[18] Kim K-H, Bae J-W (2011). Amplification methods bias metagenomic libraries of uncultured single-stranded and double-stranded DNA viruses. Appl. Environ. Microbiol..

[19] Roux S (2016). Towards quantitative viromics for both double-stranded and single-stranded DNA viruses. PeerJ.

[20] Marine R (2014). Caught in the middle with multiple displacement amplification: the myth of pooling for avoiding multiple displacement amplification bias in a metagenome. Microbiome.

[21] McNair K, Bailey BA, Edwards RA (2012). PHACTS, a computational approach to classifying the lifestyle of phages. Bioinforma. Oxf. Engl..

[22] Li J (2014). An integrated catalog of reference genes in the human gut microbiome. Nat. Biotechnol..

[23] Hugon P (2015). A comprehensive repertoire of prokaryotic species identified in human beings. Lancet Infect. Dis..

[24] Shkoporov AN (2018). ΦCrAss001 represents the most abundant bacteriophage family in the human gut and infects Bacteroides intestinalis. Nat. Commun..

[25] Yutin N (2018). Discovery of an expansive bacteriophage family that includes the most abundant viruses from the human gut. Nat. Microbiol..

[26] Waller AS (2014). Classification and quantification of bacteriophage taxa in human gut metagenomes. ISME J..

[27] Botstein D (1980). A theory of modular evolution for bacteriophages. Ann. N. Y. Acad. Sci..

[28] Cury J, Touchon M, Rocha EPC (2017). Integrative and conjugative elements and their hosts: composition, distribution and organization. Nucleic Acids Res..

[29] Bobay L-M, Rocha EPC, Touchon M (2013). The Adaptation of Temperate Bacteriophages to Their Host Genomes. Mol. Biol. Evol..

[30] Arndt D (2016). PHASTER: a better, faster version of the PHAST phage search tool. Nucleic Acids Res..

[31] Thurber RV, Haynes M, Breitbart M, Wegley L, Rohwer F (2009). Laboratory procedures to generate viral metagenomes. Nat. Protoc..

[32] Jones TH, Johns MW (2009). Improved Detection of F-Specific RNA Coliphages in Fecal Material by Extraction and Polyethylene Glycol Precipitation. Appl. Environ. Microbiol..

[33] Fouts DE (2006). Phage_Finder: Automated identification and classification of prophage regions in complete bacterial genome sequences. Nucleic Acids Res..

[34] Touchon M, Bernheim A, Rocha EP (2016). Genetic and life-history traits associated with the distribution of prophages in bacteria. ISME J..

[35] Roux S, Enault F, Hurwitz BL, Sullivan MB (2015). VirSorter: mining viral signal from microbial genomic data. PeerJ.

[36] Marcel Martin MM (2011). Cutadapt removes adapter sequences from high-throughput sequencing reads. EMBnet.journal.

[37] Modolo L, Lerat E (2015). UrQt: an efficient software for the Unsupervised Quality trimming of NGS data. BMC Bioinformatics.

[38] Crusoe MR (2015). The khmer software package: enabling efficient nucleotide sequence analysis. F1000Research.

[39] Bankevich A (2012). SPAdes: a new genome assembly algorithm and its applications to single-cell sequencing. J. Comput. Biol. J. Comput. Mol. Cell Biol..

[40] Hyatt D (2010). Prodigal: prokaryotic gene recognition and translation initiation site identification. BMC Bioinformatics.

[41] Fu L, Niu B, Zhu Z, Wu S, Li W (2012). CD-HIT: accelerated for clustering the next-generation sequencing data. Bioinformatics.

[42] Langmead B, Salzberg SL (2012). Fast gapped-read alignment with Bowtie 2. Nat. Methods.

[43] Li H (2009). The Sequence Alignment/Map format and SAMtools. Bioinforma. Oxf. Engl..

[44] McMurdie PJ, Holmes S (2014). Waste not, want not: why rarefying microbiome data is inadmissible. PLoS Comput. Biol..

[45] Oksanen, J. *et al*. *vegan: Community Ecology Package* (2018).

[46] Eddy SR (2011). Accelerated Profile HMM Searches. PLoS Comput. Biol..

[47] Rinke C (2013). Insights into the phylogeny and coding potential of microbial dark matter. Nature.

