# Supplementary material for: A simple, reproducible and cost-effective procedure to analyse gut phageome: from phage isolation to bioinformatic approach
Source: Sci Rep. 2019 Aug 5;9:11331. doi: 10.1038/s41598-019-47656-w (PMC6683287; doi:10.1038/s41598-019-47656-w)
Supplement: Supplementary file 3 — Supplementary informations [file 41598_2019_47656_MOESM3_ESM.pdf]

Supplementary information for:

A simple, reproducible and cost-effective procedure to analyse gut phageome: from phage isolation to bioinformatic approach

Camille d'Humieres[1,2,3,4*], Marie Touchon[4], Sara Dion[1], Jean Cury[4], Amine Ghozlane[5], Marc Garcia-Garcera[4], Christiane Bouchier[6], Laurence Ma[6], Erick Denamur[1,7], Eduardo P. C. Rocha[4]

[1]IAME, UMR 1137, INSERM, Université Paris Diderot, 75018, Paris, France.

[2] AP-HP, Laboratoire de Bactériologie, Hôpital Bichat, 75018 Paris, France.

[3] Ecole doctorale Frontières du vivant, Université Paris Diderot, 75013 Paris, France.

[4] Microbial Evolutionary Genomics, Institut Pasteur, CNRS, UMR3525, Paris, 75015, France.

[5] Bioinformatics and Biostatistics Hub, Institut Pasteur, C3BI, USR 3756 IP CNRS, Paris, France.

[6] BIOMICS, Institut Pasteur, 25-28 rue Dr Roux, Paris, 75015, France.

[7]AP-HP, Laboratoire de Génétique Moléculaire, Hôpital Bichat, AP-HP, Paris, France.

THIS SUPPLEMENTARY FILE CONTAINS:

SUPPLEMENTARY FIGURES 2

SUPPLEMENTARY TABLE 11

LEGENDS OF EXCEL SUPPLEMENTARY TABLES 17

LIST OF SUPPLEMENTARY REFERENCES 18

Supplementary figures

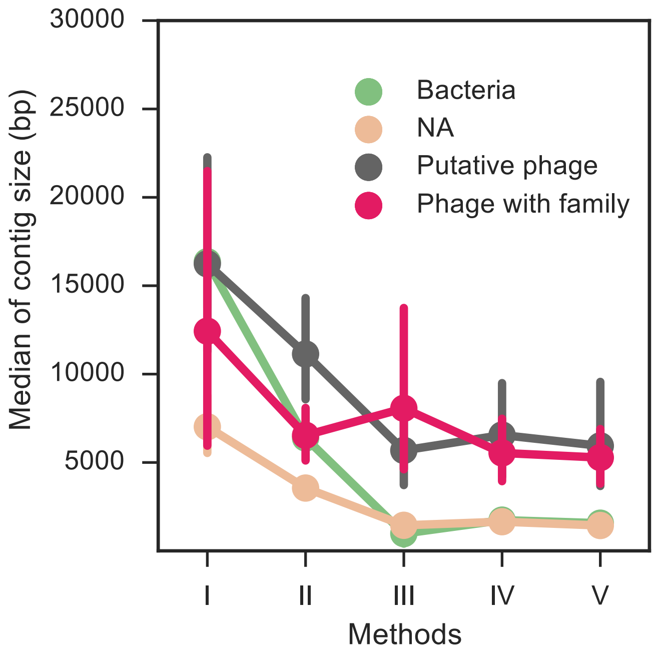

Figure S1: Median of the contigs size for each method and for each category (bacteria, "NA" and phag

NA: non-attributable

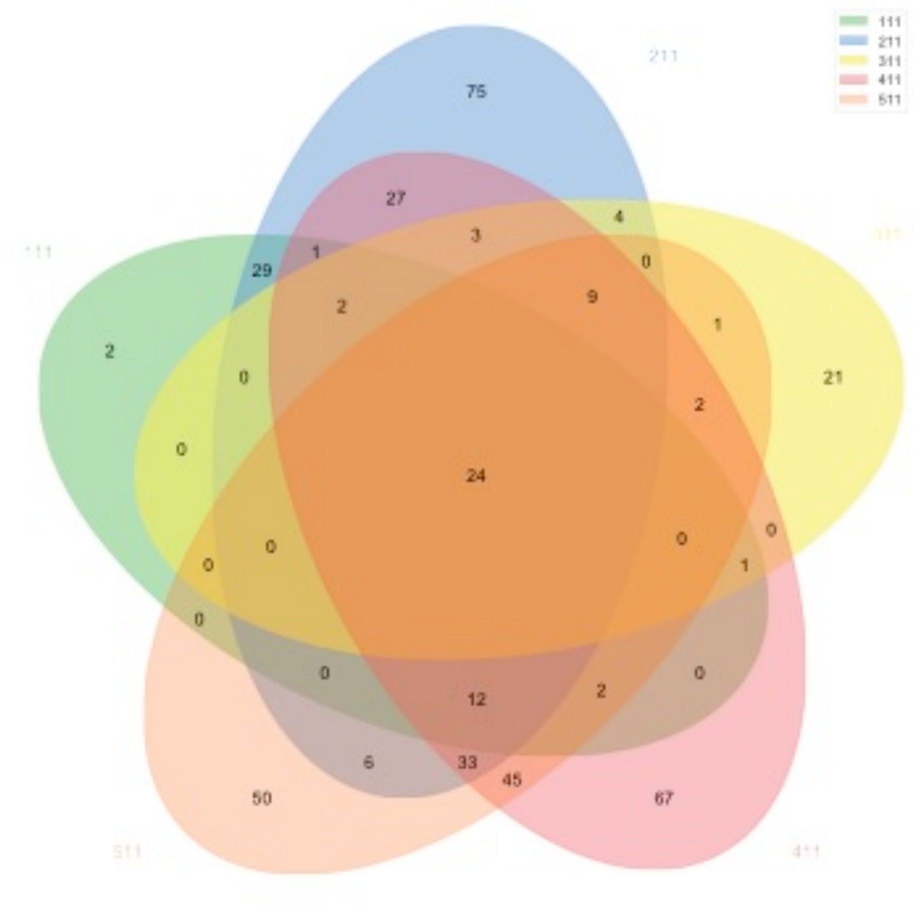

Figure S2: Venn diagram of phage clusters between each method after a local clusterisation step

Description: Venn diagram of the number of phage clusters identified for each method (only cluste where the referenced sequence is classified as "phage with family" and "putative phage"). Clusteri step was performed with CD-HIT local criteria.

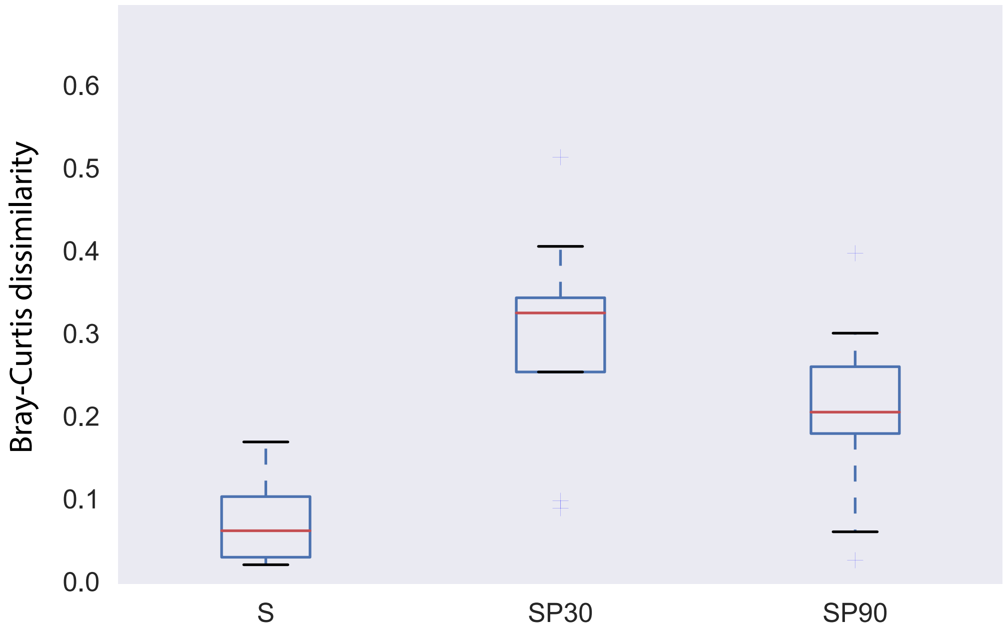

Figure S3: Box plot of the Bray Curtis dissimilarity between replicate for each methods

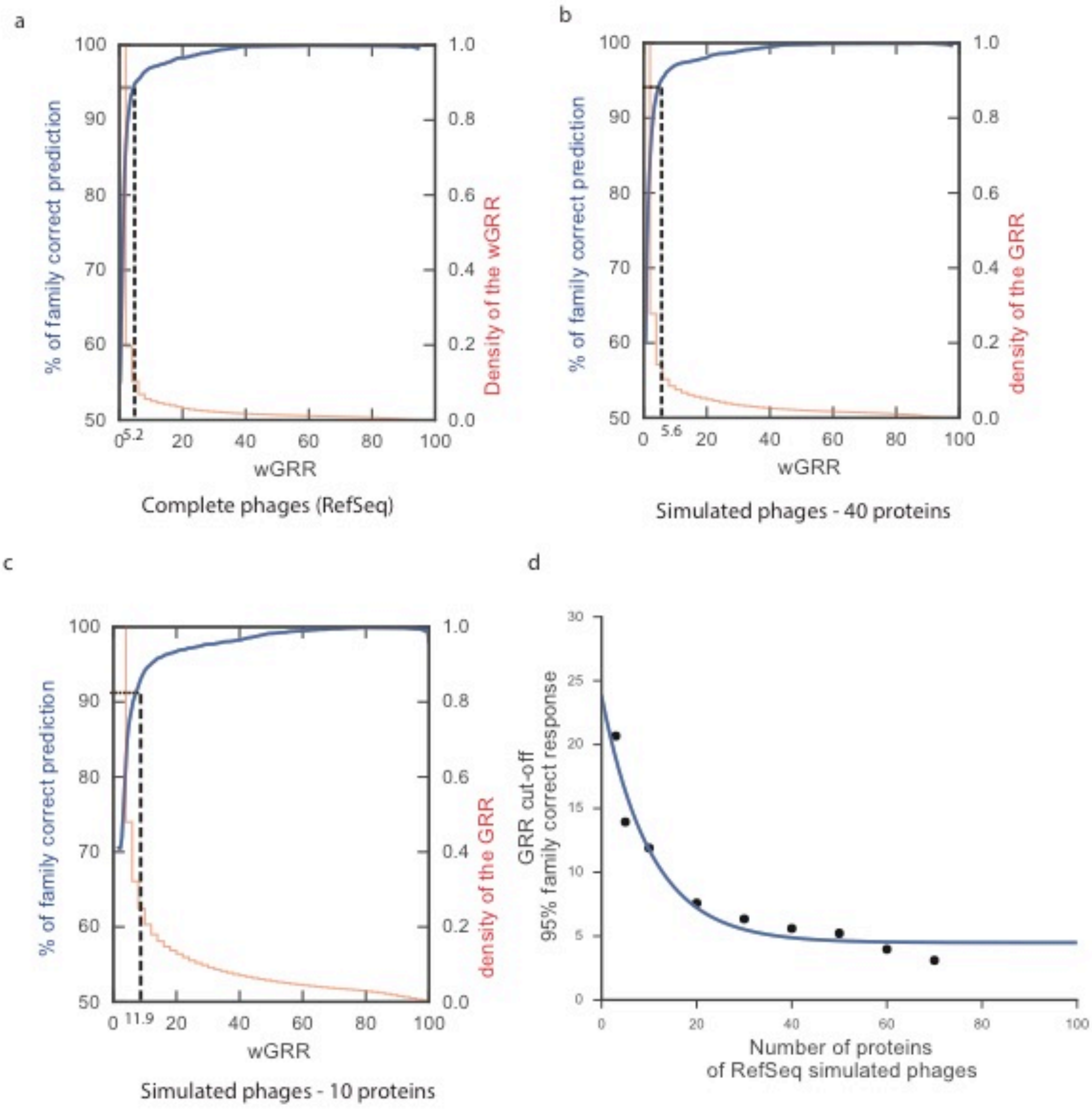

Figure S4: Determination of wGRR cut off for phage family attribution with phage Refseq database

Description: a: Evolution of the percentage of phage family correct response in terms of wGRR value for the phage of phage Refseq database. The blue line represents the proportion of phage family correct response. The red line is the decreasing cumulative density of wGRR. The dashed line represents the wGRR cut-off (5.2) for 95% correct family phage predictions. b: Evolution of the percentage of phage family correct response in terms of wGRR value between phage of phage Refseq database and a sub-sampling of this database with only part of phage of 40 proteins. The dashed line represents the wGRR cut-

off (5.6) for 95% correct family phage predictions. c: Evolution of the percentage of phage family correct response in terms of wGRR value between phage of phage Refseq database and a sub-sampling of this database with only part of phage of 10 proteins. The dashed line represents the wGRR cut-off (11.6) for 95% correct family phage predictions. d: wGRR cut-off for a phage family correct response in terms of size of simulated phages. This curve fit is 0.95 with this equation $Y = 19.33 \times e^{-0.098X} + 4.49$ where Y is the wGRR cut-off and X the number of proteins of the simulated phages (or of the contigs).

		Virsorter putative phage	16S DNA bacterial	HMM Markers bacterial	HMM markers phage	NCBI
GRR phage family	506	288	0	6	274	17B* – 14V*
Virsorter putative phage		663	0	11	329	19B* – 11V*
16S DNA Bacterial			6	0	0	0
HMM markers bacterial				443	26	49B
HMM markers phage					444	34B* – 2V* – 1E*
NCBI						506

* B: Bacterial contig, V: Virus contig, E: Eukaryota contig

Figure S5: Details of the repartition of the 6055 contigs in the ABC-Reference-Contig-Catalog with t[illegible] decision algorithm in Figure 1a.

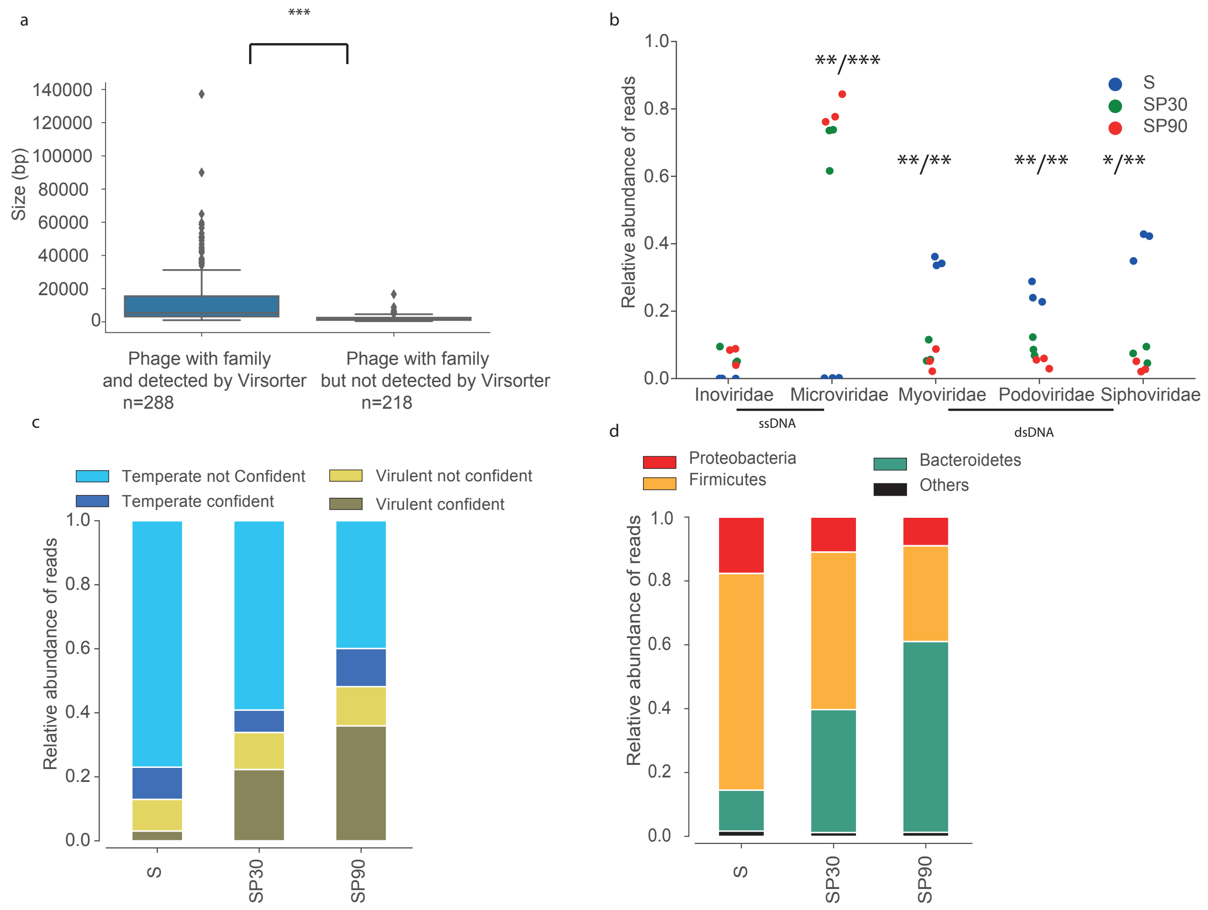

Figure S6: Re-analysis of the contigs of the healthy volunteers' (AA, BB, CC) samples, removing the 218 contigs classified as "Phage with family" but not detected by Virsorter. (a): Boxplot of the size (bp) of the 508 contigs classified as "Phage with family" and divided into two groups (detected or not by Virsorter as phages, ***p<0.001, Wilcoxon test) (b): Plot of the relative abundance of reads for each phage family, conciliating only the 208 contigs classified as "Phage with family" and detected by Virsorter. For microviridae, myoviridae and siphoviridae the relative abundance of reads are significantly different between MDA protocoles (SP30, SP90) and no MDA protocoles (S), as assessed by paired t-tests (*<0.05, **<0.01, ***<0.001). (c) Bar plots of the relative abundance of the reads mapping the contigs of a certain lifestyle, removing the 218 contigs classified as "Phage with family" but not detected by Virsorter. (d) Bar plots of the relative abundance of the reads mapping the

contigs of a certain host phyla, removing the 218 contigs classified as “Phage with family” but not detected by Virsorter. NA: non-attributable.

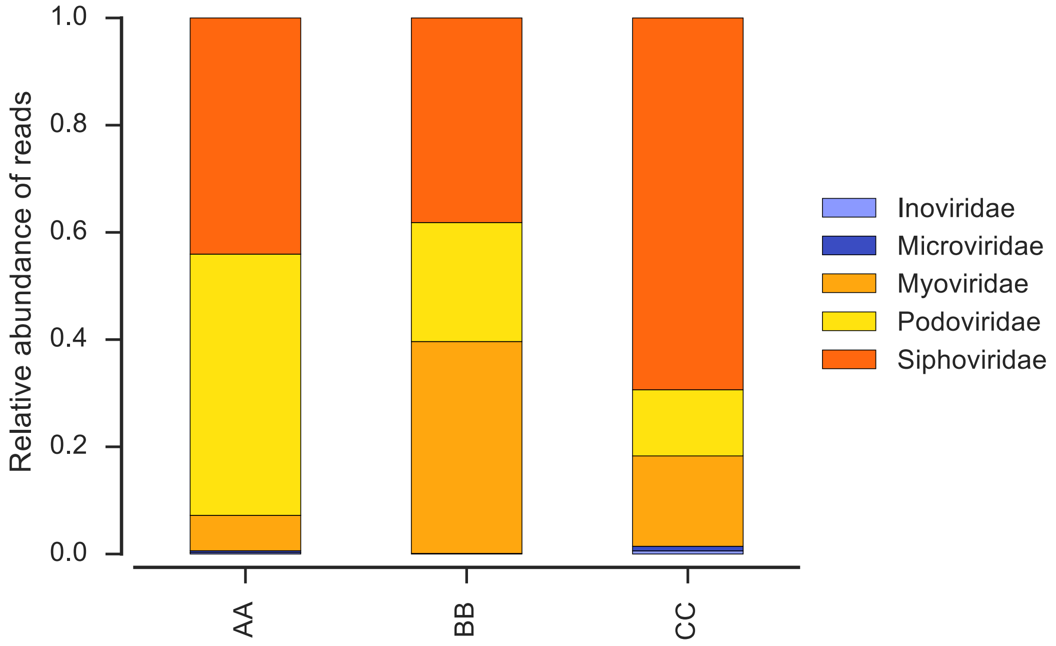

Figure S7: Bar plots of the relative abundance of reads belonging to “phage family” contig categories the method S without MDA in the three healthy volunteers (AA, BB, CC)

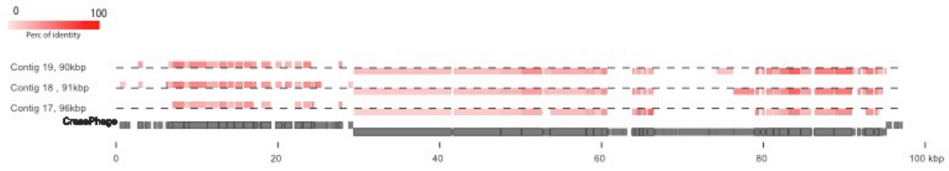

Figure S8: Details of the three contigs of the crAssphage like.

Description: Three contigs of the ABC-Reference-Contig-Catalog are related to the crAssphage, contigs numbers 17, 18 and 19 with respectively a wGRR of 19, 27 and 26. Each red little box represents a homology between a protein of the contig and a protein of the crassphage. The intensity of the red colour is proportional to the percentage of identity between the 2 proteins.

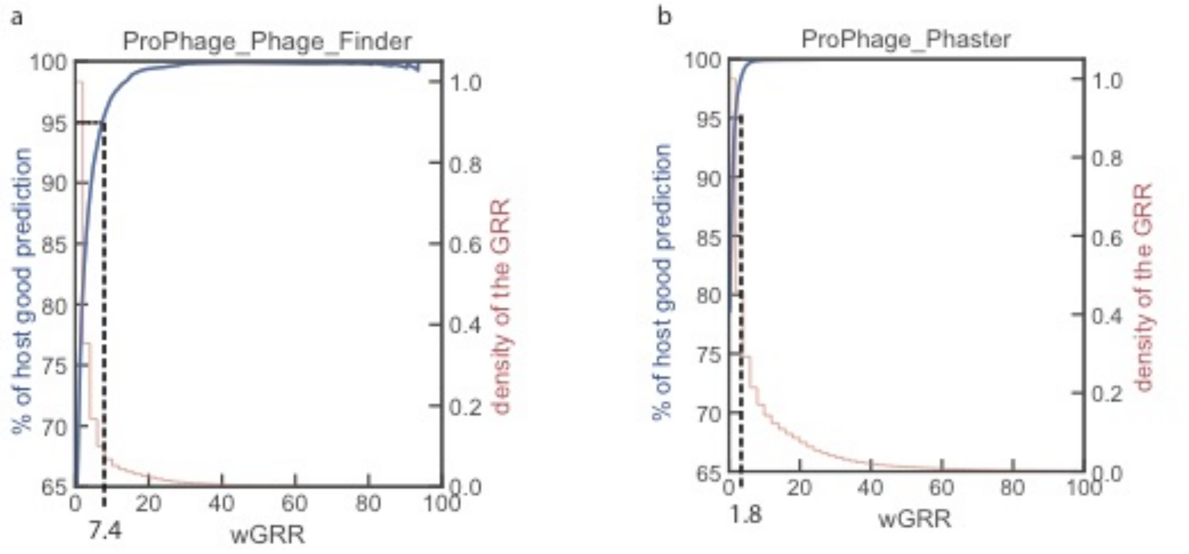

Figure S9: Comparison of the evolution of the percentage of host prediction correct response in terms of wGRR value for the prophage of bacteria Refseq database found with

two different software of prophages prediction: an old one: Phage finder and a new one: Phaster.

Description: (a) We identified 9927 prophages in the chromosomes of complete bacterial genomes from GenBank Refseq using Phage Finder v4.6. Many of these prophages were very similar. We clustered them with a wGRR cut off of 95% and we took a representative of each cluster (6426 phages). In the graph the blue line represents the proportion of host correct response. The red line is the decreasing cumulative density of wGRR. The dashed line represents the wGRR cut-off (7.4) for 95% correct host predictions. (b) We identified 11239 prophages in the chromosomes of complete bacterial genomes from GenBank Refseq using Phaster (online version, only intact prophages were selected). In order to reduce the dataset we clustered them with a wGRR cut off of 95% and we took a representative of each cluster (5786 phages). In the graph the blue line represents the proportion of host correct response. The red line is the decreasing cumulative density of wGRR. The dashed line represents the wGRR cut-off (1.8) for 95% correct host predictions

SUPPLEMENTARY TABLE

Method	Use of MDA	No MDA
Filtration	[1–5]	
Filtration + CsCl[a] gradient	[6–10]	[11–1314,15]
Filtration + Ultrafiltration	[16]	[17]

[a] CsCl: Cesium chloride

Table S1: Literature overview of the methods for phage isolation in faeces and the utilisation of tl

multiple displacement amplification (MDA) process

Name	Number of reads	Number of contigs	Number of contigs (at least 3 ORFs)	Mean of the contig size (bp)	Median of the contig size (bp)
AA_0 IV LC	1 183 870	42 584	952	2 789	1 451
AA_0 IV HC	87 148 950	68 8041	15 656	4 956	1 678

Table S3: Comparing statistic between sample (AA_0) extracted with method IV at a low coverage (LC) and at a high coverage (HC)

Name	Number of reads
**IV_HC**	87 148 950
IV_90p	78 429 610
IV_80p	69 724 386
IV_70p	61 005 126
IV_60p	52 287 638
IV_50p	43 574 052
IV_40p	34 846 898
IV_30p	26 143 282
IV_20p	17 422 864
IV_10p	8 713 526
IV_1p	870 448
**IV_LC**	1 183 870

Table S5: Number of reads of the 10 simulated datasets and of the two observed datasets IV_HC (hi[illegible] coverage) and IV_LC (low coverage)

Database	Information	α	β	γ	R^2
RefSeq Phage	Family	19.33	-0.098	4.49	0.95
	Host	13.64	-0.18	7.34	0.96
	Lifestyle	10.75	-0.23	0.44	0.98
Prophage HMP	Host	28.78	-0.32	13.98	0.95
Prophage RefSeq Bacteria	Host	30.30	-0.22	5.26	0.99

Table S6: Summary of the different values (α, β and γ) of the equation type $Y = \alpha \times e^{-\beta X} + \gamma$ where is the wGRR cut-off and X is the number of proteins of the simulated phage (or of the contig) for ea databases and for each information. The last column "R^2" represents the fit of the regression line

a

Categories	Number of contigs
No attribution	3489
Putative PHAGE	1122
Bacterial Contamination	914
PHAGE with family	506
Unknown contamination	22
Eukaryota Contamination	2
Archaea Contamination	1

b

Categories	Number of contigs
No attribution	705
Temperate-Confident	603
Temperate-Not-Confident	160
Lytic-Confident	96
Lytic-Not-Confident	54

c

Categories	Number of contigs
Firmicutes	818
No attribution	544
Bacteroidetes	144
Proteobacteria	20
Actinobacteria	27
Fusobacteria	9
Synergistetes	3
Acidobacteria	1
Deinococcus-Thermus	2
Crenarchaeota	1

Table S7: Exploration of the 6,056 contigs found in healthy volunteers (AA, BB, CC) samples

Description: a: Table of description of the different categories of contigs

b: Table of description of the different categories of lifestyle of contigs,

c: Table of description of the different bacteria host (phyla) of contigs.

LEGENDS OF EXCEL SUPPLEMENTARY TABLES

Table S2

File format: XLS file

Title: Tab number 1: HMM profile, from PFAM (30.0) and TIGRFAM (15.), phage specific with a keyword like "capsid, core, portal, protease, replication, tails" and HMM profile bacteria specific. Tab number 2: Details of the HMM profile of the bacterial genes.

Table S4

File format: XLS file

Title: Exploration of different CD-HIT clustering settings on the two dataset: 411 low coverage (LC) datas and 411 high coverage (HC) dataset.

LIST OF SUPPLEMENTARY REFERENCES

1. Lim, E. S. *et al.* Early life dynamics of the human gut virome and bacterial microbiome in infants. *Nat. Med.* **21**, 1228–1234 (2015).
2. Zuo, T. *et al.* Bacteriophage transfer during faecal microbiota transplantation in Clostridium difficile infection is associated with treatment outcome. *Gut* **67**, 634–643 (2018).
3. Conceição-Neto, N. *et al.* Modular approach to customise sample preparation procedures for viral metagenomics: a reproducible protocol for virome analysis. *Sci. Rep.* **5**, 16532 (2015).
4. Reyes, A. *et al.* Gut DNA viromes of Malawian twins discordant for severe acute malnutrition. *Proc. Natl. Acad. Sci. U. S. A.* **112**, 11941–11946 (2015).
5. Kim, K.-H. & Bae, J.-W. Amplification methods bias metagenomic libraries of uncultured single-stranded and double-stranded DNA viruses. *Appl. Environ. Microbiol.* **77**, 7663–7668 (2011).
6. Reyes, A. *et al.* Viruses in the faecal microbiota of monozygotic twins and their mothers. *Nature* **466**, 334–338 (2010).
7. Minot, S. *et al.* The human gut virome: Inter-individual variation and dynamic response to diet. *Genome Res.* **21**, 1616–1625 (2011).
8. Norman, J. M. *et al.* Disease-Specific Alterations in the Enteric Virome in Inflammatory Bowel Disease. *Cell* **160**, 447–460 (2015).
9. Modi, S. R., Lee, H. H., Spina, C. S. & Collins, J. J. Antibiotic treatment expands the resistance reservoir and ecological network of the phage metagenome. *Nature* **499**, 219–222 (2013).

10. Abeles, S. R., Ly, M., Santiago-Rodriguez, T. M. & Pride, D. T. Effects of Long Term Antibiotic Therapy on Human Oral and Fecal Viromes. *PloS One* **10**, e0134941 (2015).
11. Breitbart, M. *et al.* Viral diversity and dynamics in an infant gut. *Res. Microbiol.* **159**, 367–373 (2008).
12. Manrique, P. *et al.* Healthy human gut phageome. *Proc. Natl. Acad. Sci. U. S. A.* **113**, 10400–10405 (2016).
13. Allen, H. K. *et al.* Antibiotics in feed induce prophages in swine fecal microbiomes. *mBio* **2**, (2011).
14. Górska, A. *et al.* Dynamics of the human gut phageome during antibiotic treatment. *Comput. Biol. Chem.* **74**, 420–427 (2018).
15. Johnson, T. A. *et al.* The In-Feed Antibiotic Carbadox Induces Phage Gene Transcription in the Swine Gut Microbiome. *mBio* **8**, (2017).
16. Minot, S. *et al.* Rapid evolution of the human gut virome. *Proc. Natl. Acad. Sci.* **110**, 12450–12455 (2013).
17. Fernández-Orth, D. *et al.* Faecal phageome of healthy individuals: presence of antibiotic resistance genes and variations caused by ciprofloxacin treatment. *J. Antimicrob. Chemother.* **74**, 854–864 (2019).
